# Necessity of the Needs Map in the Service Design for Smart Cities

**DOI:** 10.3389/fpsyg.2020.00202

**Published:** 2020-02-25

**Authors:** Seyun An, Sungwhan Kim, Soyeon Kim

**Affiliations:** ^1^Department of Industrial Design, College of Construction Environment and Design, Hanbat National University, Daejeon, South Korea; ^2^Institute for Basic Science, College of Engineering, Hanbat National University, Daejeon, South Korea; ^3^User Centered Smart Cities Research Cluster, Hanbat National University, Daejeon, South Korea

**Keywords:** smart city, needs map, service design, living lab, user centered design

## Abstract

Recently, the issue of how to involve actual service users in the service designs for Smart cities has grown in importance, as this is a key factor in determining the serviceability and sustainability of developed services. Reckless participation of users can make the service design process inefficient and cause a cost increase. So, in this study, we propose the needs map which can be applied to various stages of service development and help service designers to make efficient and reasonable decisions without being too time-consuming. The proposed needs map is a schematization of theoretical concepts for Smart city planning in accordance with the framework of service-technology-infrastructure-management (STIM). Through the needs map, various urban problems, statistical data, and users' needs in a smart city are classified and analyzed concept by concept, and these focused analysis results suggest a proper direction to researchers in the service development plan. To demonstrate experimentally the feasibility of the needs map, we applied it in practice to a service design for the Smart city experience zone located in Daejeon, South Korea.

## Introduction

### Study Background and Objective

The smart city is a future high-tech city in which various information services can be provided through devices whenever and wherever by using Information Communication Technology (ICT), and is a concept that has gradually evolved from Information Cities and U-Cities. When the term “Smart city” is mentioned, rather than a simple compound of two words “Smart” and “City” in some sense it means a process of making existing cities smart. PAS 180 announced by BSI (British Standards Institution) defines “Smart city” as the effective integration of physical, digital, and human systems in the built environment to deliver a sustainable, prosperous, and inclusive future for its citizens (British Standards Institution BSI, 2014).

Recently, the global interest in the Smart city is growing. In India, the Modi Government that was inaugurated in 2014 declared its Smart city policy as one of its core missions, and it designated 100 mart cities nationwide as of 2018. China also announced the construction plan of 500 smart cities until 2020. As of the end of 2017, about 500 smart cities were under construction in China, and they occupied 50% of smart cities under construction in the world. Among them, the Chinese government selected officially 277 cities as a model of smart sights. In 2019, the Korean Ministry of Land, Infrastructure, & Transport (MOLIT) announced the “3rd Comprehensive Plan on Smart Cities (2019~2023)” which integrated policies and projects from government departments. It is a mid-to long-term master plan for the construction and proliferation of smart cities and the creation of innovative ecosystems, and the enhancement of global initiatives. In this period, the Korean Ministry of Public Administration & Security (MOPAS) also held a Smart City Joint Workshop with the World Smart Sustainable Cities Organization (WeGO), the World Bank, and the International Telecommunications Union (ITU), and 19 countries including India, Malaysia, and Kyrgyzstan participated in the workshop. Furthermore, local authorities and central government departments are continuing the efforts for new projects for smart cities.

Technology adoption is indispensable in developing Smart cities and there are two approaches to applying cutting-edge technologies to making existing cities smart. One is the tech-driven method (TDM) and the other is the human-driven method (HDM) (Kummitha, 2018). In TDM, under the leading of government officials or policy makers, new services for Smart cities are developed by service providers or private companies equipped with technologies, in many cases for their business. Meanwhile, HDM requests the active participation of the citizens so that technologies and services for them are developed according to their actual needs. In many development processes of Smart cities in the world, however, TDM has been the most common method used. An enormous number of innovative services for Smart cities have been proposed by researchers from all over the world, with vague hopes that these services will help to solve various problems confronting large cities (Kummitha, 2018). However, only a small number of them have been put to practical use and the rest have disappeared from (local) communities right after their commodification, due to their impracticality to communities. This has occurred because researchers did not consider what is actually needed by the cities, but simply imitated the existing products to focus on and apply specific technologies (Oktaria and Smart City Services, 2017). Even though Korea has an advanced Information & Communication Technology (ICT) environment, developing its Smart cities also leaned toward fragmentary technological developments.

In recent years, the issue of how to involve actual service users in the service designs for Smart cities has grown in importance, since this is an important factor in determining the serviceability and sustainability of developed services (Kummitha, 2019). In the service design for Smart cities, user involvement is required in each stage of finding issues, defining terminologies, developing services, and evaluating results, although reckless participation of users can make the service design process inefficient and cause a cost increase. For instance, Living Lab is an open methodology service aimed at addressing and confronting problems in a (local) community through the direct participation of various interested parties (Living Lab Guide, 2019). Experts or facilitators encourage discussion between (or with) participants in Living Lab meetings, but in practical Living Lab cases the individuality and diversity of people's experiences and preferences detracts from the Lab's ability to efficiently draw useful insights. In consideration of limited budget and time, the determination of the scope or direction of discussion before direct user participation in a Living Lab prevents rambling discussion and keeps participants focused. In fact, if the issues are clear and do not cause major conflicts of interests, it may be enough for experts to determine issues and derive solutions from indirect user participation (such as questionnaires, various statistical data, etc.). This suggests the necessity of a new methodology which should be efficient at user-involvement and have a practical application in real cities. In this paper, we want to develop a new framework through which miscellaneous needs, interests, or requirements from users and citizens are categorized and interpreted in the functional terminology for understanding real cities. This indirect method for user involvement may help, e.g., designers to have definite insights on users' needs, and these insights can be applied to the service development.

To understand the urban structure and design the urban space, many structural factors and functional concepts have been proposed by previous researches and widely accepted. Given the service design or space planning for Smart cities, user involvement without consideration of these characteristic features through which the system of cities can be understood results in vacuous debates, and also does not guarantee the sustainable serviceability of developed services. So, we are interested in devising a new method based on these theoretical features to provide a fundamental yardstick for service designers to use at each stage of the service design process for smart cities. In this paper, we propose the needs map as a schematization of theoretical concepts for Smart city planning in accordance with the framework of the service-technology-infrastructure-management (STIM). This needs map can help designers to analyze and confront urban issues from various essential perspectives and find some insight either in the Living Lab process or before it starts. Through such common insight about interested issues, co-creation between users, service designers, government officials, etc. would be possible. Also, the users' needs within various social fields with urban issues can be closely understood through the needs map and reflected in the service development. That is to say, the proposed needs map is a methodology not for user participation but for smart city-tailored filtering and efficient utilization of a variety of users' needs in actual cities. To demonstrate the feasibility of the proposed needs map, we will provide an application of a Needs Map to the service design for the Smart city experience zone located in Daejeon, South Korea.

### Study Method and Scope

In this study, we will form the conceptual framework for the needs map from the viewpoint of services for the Smart city, and it will be utilized to develop services for the Smart city experience zone to verify its necessity for the Smart city service design. The study method and scope are organized as follows.

We will take three steps in this study. In the first step, key concepts for the needs map framework are considered through the literature study. In particular, we will seek to understand essential notions or entities for the Smart city service design using previous works that contain ISO SMART CITY CONCEPT MODEL GUIDELINE, and by consulting experts. In the second step, the needs map, which will consist of four layers, will have key entities appropriately located on it, with each layer being divided into four sides of service, technology, infrastructure, and management. This needs map provides useful information about what should be considered in designing specific services. Finally, the proposed needs map will be experimentally applied to service designs for the smart city experience zone to demonstrate the usability or feasibility of the needs map.

Indeed, the needs map is quite utilizable in the discovery step of the service design process based on Design Thinking, in which user's surveys and studies on the situation are performed. Service completeness can also be improved by double-checking concepts missed in the service realization process. By using the needs map, researchers can thoroughly investigate extensive urban issues or users' needs in a relatively short time through cooperation with interested parties. Eventually, the needs map will help planners to survey vastly diverse urban experiences and develop sustainable services to reflect the purpose of the user-centered smart city.

## Explanation for the Needs Map

### STIM Framework of the Needs Map

The eventual purpose of this study is to develop services suitable for Smart cities by finding refined needs, not from the simple classification of users and operation managers, but from the analysis of a variety of raw needs from interested parties. We hope that the proposed needs map will play such a methodological role. We organized the needs map on the basis of the STIM framework. U-City emphasized technology-based infrastructure planning (Lee and Leem, 2008), but Smart city is centered around space planning and being a people-oriented service (An, 2016; Korea Agency for Infrastructure Technology Advancement, 2017b). This STIM notion has evolved into the ICTs-based U-city which focused on service and technology. Through this paper, the “user-centered” concept is assimilated into the STIM framework, and eventually into the needs map.

For the needs map, we take the perspective of Service-Technology-Infrastructure-Management for the Smart city design. Service is based on the various needs of users themselves. To detect the human motion and state—since Technology should support two-way communication with citizens as users of the urban space and management—various technologies such as cameras, sensing technologies, interface technologies, GPS, and telecommunication are required. Specifically, Infrastructure includes physical facilities equipped with technologies just mentioned to realize services into the real space. On the other hand, Management refers not just to service operation, but to the concreteness and realizability of the business model for sustainable services. In the STIM framework, Infrastructure and Management are related for function, Service and Management for contents, Infrastructure and Technology for interface, and Service and Technology for interaction. To enable these to be embodied in real cities, SW, HW, and System designs are required.

### Four Layers on the Needs Map

**Figure 1:**
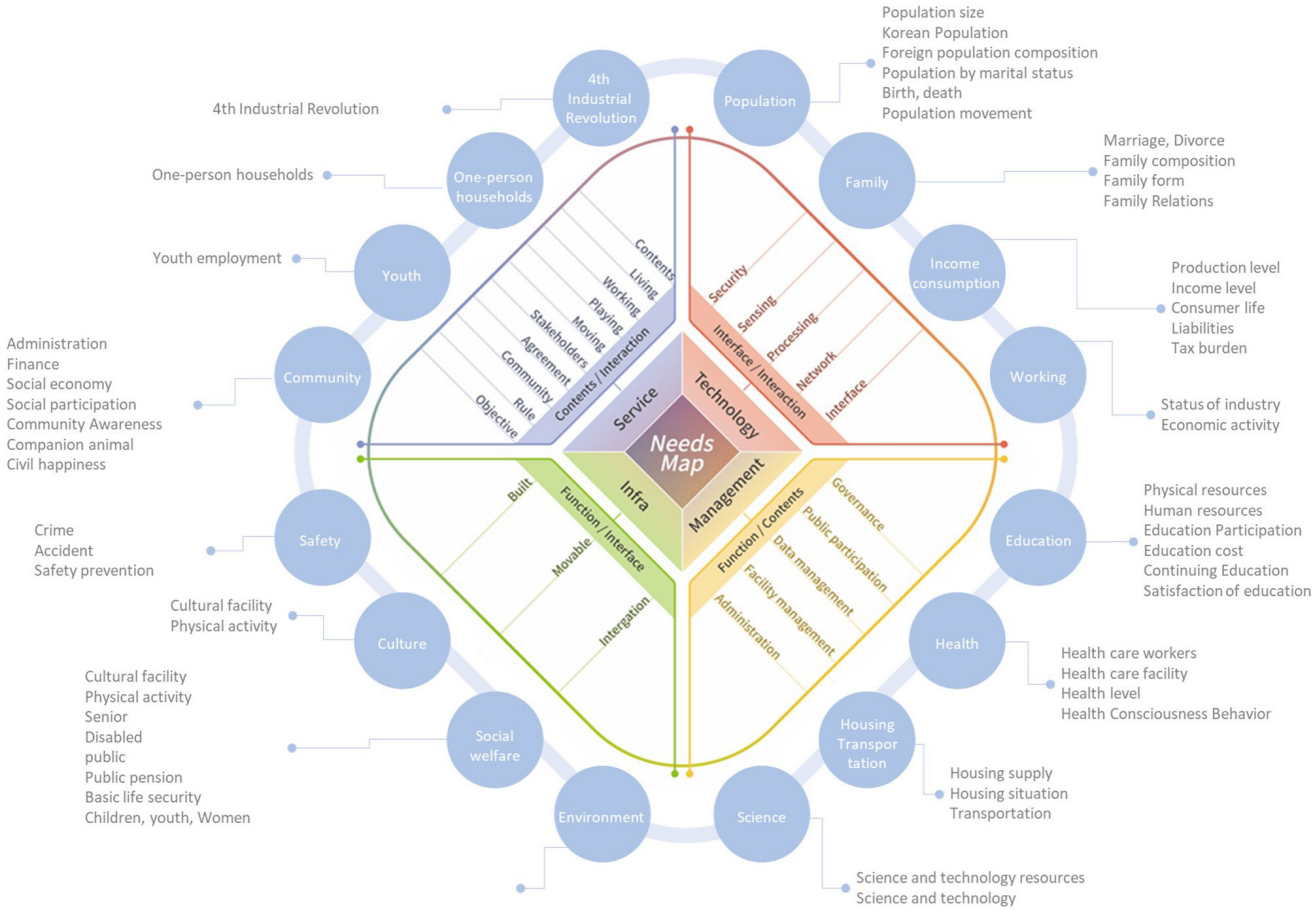
Needs map.

The needs map is composed of four rectangular layers based on the STIM framework. In fact, users' needs can be analyzed through various forms (for instance, questionnaires, user behaviors, visit frequency to websites, etc.). In order to reflect these needs to the service design development for Smart cities, they should be interpreted and categorized in a standard manner. Going outward from the center, the terms on the tiers become specific and detailed in the perspective of the STIM embedded model. For the needs map shown in (Figure 1), a round multi-layer structure is used to emphasize the hierarchical relation between layers and the horizontal one between terminologies on a layer. The explanation of the layers follows.

Layer 1 is based on the STIM embedded design model. It is a promotion process for planning, designing, and operating a Smart city and is a multi-layer design model with Service-Technology-Infrastructure-Management layers which are on four rectangular sides of Layer 1, respectively.

Layer 2 is for the functional aspect of the STIM embedded design model. The service sector of Layer 2 is composed of Contents and Interaction. The technology sector represents Interface and Interaction. The infrastructure sector includes Function and Interface. The management sector consists of Function and Contents. Entities on Layer 2 are defined as follows: Contents are a variety of information and details realized ultimately in the perspective of the software; Interaction is the entity for realizing the user-centered design in terms of interaction between service environment and user; and Interface is the medium and protocol for communication between thing to thing or thing to human, and should be considered for practical realization of technology models for the Smart city. Function is to help physical Infrastructure and systematic Management to play a consistent and efficient role.

While Layer 2 is for functional approach of the STIM model, Layer 3 represents practical entities for design and realization in the real space. Each entity on Layer 3 can be used as the detailed goal which should be solved to realize the service.

Layer 4 is composed of the data indicators that will be used to draw miscellaneous insights required for the service development of the Smart city. Sixteen statistical indicators and their details appear on Layer 4. In fact, these indicators are used in national annual surveys as statistical social indicators. Each indicator will become a region and survey subject to which practical services for the Smart city should be provided. For instance, the field of residential transportation is an important indicator which the future housing or urban model should provide as a service for the Smart city, and furthermore the supply and demand of housing, the residential status, the convenience of transportation, etc., should be checked.

### Functional Entities on the Layers

In this study, the needs map is defined as a visualized output on which essential entities for the Smart city service are linked into the STIM framework according to their functional meanings. In fact, before obtaining these functional entities on the layers, many important concepts for the Smart city were discussed and extracted through several expert advisory councils in line with the advent of the Smart city over the U-city (see Table 1). To derive fundamental concepts for Smart cities and test beds, we held workshops and conferences for experts in which discussions between experts were made through brainstorming methods based on design thinking^1^. In addition, an advisory conference of relevant experts was held to better understand the experience-type test bed for the Smart city and for establishing its design process. Experts for the conference included professors and chief researchers from the fields of urban engineering, construction and design, etc. In the advisory conference, contents for the service, technology, infrastructure, and operation fields were discussed to determine core elements and directions of the Smart city.

**Table 1:** Expert advisory councils to find fundamental concepts for the smart city.

**Category**	**Council/Conference**	**Experts**	**Extracted keywords**
Goal and direction settings	Smart city key elements and direction (2017.4.5)	- Creative technology management - Urban engineering	Governance, infrastructure integration, Service innovation, cooperative partnerships, Urban innovation, Intelligence and sustainability, Urban Openness
Service	Smart well user-centered smart welfare direction and strategy (2017.9.28)	- Korea facility management association - Industrial design	User, Living lab, Lifecycle, Diversity, behavior
Technology	Building energy efficiency improvement technology and evaluation (2017.6.19)	- Urban engineering - Facility engineering - Industrial design information and - Communication engineering	Energy, modeling, process, sensor, integration, efficiency, data, existing buildings, new buildings, certification assessment
Infra	Spatial big data-based smart safe City (2017.7.24)	- Urban engineering	Big data, Safety, Security, Prevention
Management	Big Data/ IOT + Cognitive Computing/Simulation (2017.5.29)	- Electronics and telecommunications - Research institute - Future strategy research institute - Industrial design - Urban engineering	Big data, Collaboration, Collective intelligence, Analysis, Linkage, Simulation, Strategy, Policy, Law, Institution

In this paper, on the basis of these fundamental concepts for the Smart city, we discreetly selected functional entities through careful literature studies which are arranged on the layers in accordance with their practical functions. These entities are chosen to accomplish the purpose of the user-centered Smart city, and so their physical and systematic perspectives are continuously deliberated to incarnate them in sustainable services on (smart city) experience zones. Quantitative social indicators or statistical indicators also are considered to address various urban issues. These entities are distributed on layers of the needs map which form in stratum. Entities on a higher layer are more specific than those on a lower one.

To reflect the various needs of users, the service sector in the needs map can show reasons or purposes for why users require and use the service. So the service sector should contain contents, their role and goal, and communication and cooperation with various related parties. In particular, contents for the Smart city should include “living” (for supporting the living convenience and safe life of the citizens), “work” (for activating the local economy of production and enhancing the work efficiency of the related parties), “entertainment” (for activating citizen participation in the local community by specializing local cultural asset), and “mobility” (for securing the convenience, approach, and comfort in pedestrian and transportation methods).

As described earlier, technology supports two-way communication with citizens as users in the urban space and management. So, contents for the technology sector should include sensing and interface technologies for motion detection and network processing and security for information exchange.

Infrastructure includes physical facilities to realize services into the real space, and so the infrastructure sector should consider contents which are related to terms such as “built,” “movable,” and “variable.”

Management implies the concreteness and realizability of the business model for sustainable services, and so the management sector should include contents such as governance establishment, private cooperation, public participation, facility operation-management, and maintenance of contents and information.

Table 2 shows the detailed explanation of structural elements of the needs map.

**Table 2:** Explanation of functional entities on the needs map.

**Category**	**Definition**	**Explanation**
Layer 1	STIM embedded design model (Lee and Leem, 2008)	Multilayer service-technology-infrastructure-management design model		
Layer 2	Functional approach: Functional approach of STIM embedded design model (An and Kim, 2016)	Contents, Interaction, Function, Interface		
Layer 3	Spatial Planning: Practical entities for design (Lee et al., 2008, 2009, 2017; Komninos et al., 2011; Falconer and Mitchell, 2012; Lazaroiu and Roscia, 2012; Korea Agency for Infrastructure Technology Advancement, 2014, 2015, 2016, 2017a,b, 2018; An et al., 2016, 2019; Lee and Leem, 2016; Snow et al., 2016; Datta and Roy, 2017; ISO/IEC30182, 2017a; Kim and An, 2017a,b, 2018; Park et al., 2018; Living Lab Guide, 2019, User Centered Smart Cities Research Cluster)	Contents	Contents	Service experience type
			Living	Life-related
			Working	Work-related
			Playing	Play related
			Moving	Move-related
		Stakeholders		Various stakeholders related to service support and benefit from service creation to utilization
		Agreement		Negotiation agreement between various stakeholders regarding user behavior in the service process
		Community		Individuals and organizations that share common goals or characteristics
		Rule		Explicit or understood regulations or principles governing behavior or procedures within specific areas of activity
		Objective		Requirements of Diverse Stakeholders
		Security		Information security
		Sensing		Sensor type
		Processing		Build process
		Network		Advanced network
		Interface		Communication media and protocol
		Built		Fixed infrastructure
		Movable		Portable infrastructure
		Integration		Variable infrastructure
		Governance		Public and private cooperative structure that enables cooperation
		Public participation		Participation design for user-centered service implementation
		Data management		Data management for efficient smart city service
		Facility management		Comprehensive system for efficient operation management of smart city information
		Administration		System for effective governance
Layer 4	Smart City Experience: the data indicators to draw miscellaneous insights (ISO/IEC30182, 2017b)	Social index survey statistics conducted by nationwide survey every year, 16 indicators and detailed indicators		

### Mathematical Explanation for the Needs Map

The needs map in Figure 1 provides a way to understand users or citizens' needs for service developments in cities. Speaking in detail, all behaviors which users perform in a city are analyzed and grouped into needs through the needs map, with individual services developed from extracted user needs. For example, in the next section, image analysis devices were used for the behavior analysis of the users visiting the Doan District which is a new town in Yuseong-gu, Daejeon, South Korea, and user needs were discovered from the analysis result. In this example, the amount of data was relatively small and was able to be manually dealt with. This local study shows effectively the usefulness of the needs map in service developments for Smart cities. However, to apply the needs map to Smart cites as a whole requires planners to maneuver the immense amount of data which is constantly updated. Needs from variously interested persons who are men, women, residents, visitors, officials, etc. are also complicated and extensive. This shows another reason why the needs map is necessary. The needs map proposes a framework model through which user behaviors are classified, managed, and utilized for needs extraction and further service developments for smart cities.

**Figure 2:**
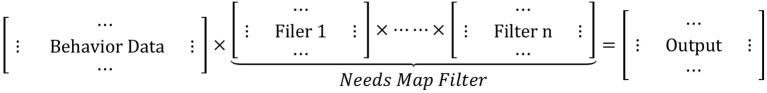
Data classification process using filters.

The classification of such large-scale data requires several filtering processes of data sets. In algorithms for Big Data Analysis, a filter or kernel is given in the form of a matrix and, in fact, the filtering process is a convolution of the filter with a data set. So to design filters appropriate to the purpose is very important in data classification. A needs map should permeate these filter matrixes. This filter design for the need map will be one of our future studies (see Figure 2).

## Experimental Application of the Needs Map To the Smart City Experience Zone

In this section, we show the feasibility of the needs map in Figure 1 to develop services for a Smart city experience zone. To do so, we take the following three steps. First, by using statistical social indicators we obtain overall spatial information related to entities on Layer 4. This spatial information helps to understand the current status of the experience zone. In particular, the environment and security issues in the experience zone drawing high interests of users are intensively discussed. Second, based on the pabulum obtained in the first step, a place in the experience zone to which services will be practically provided is chosen and perambulated. The reconnaissance of the chosen region based on the needs map helps to evaluate the adaptability of the practical space design and the service application. We obtain baseline data from the investigation of physical environment and people's behaviors in the reconnaissance for service developments. Third, the purpose and direction for services which will be provided to the experience zone are set up. Those services are practically realized in the chosen space.

### The 1st Step of the Needs Map Application: to Understand the Experience Zone Through Layer 4

In this step, as services developed for smart cities are intrinsically used by a large number of citizens in public places, the environment issue in the experience zone is considered. According to statistics of social indicators released by Daejeon Metropolitan City (2018) 25.1% of its residents consider the garbage problem as something that should be quickly addressed, with 22.9% reporting on the air pollution and stench problem. Furthermore, “the destruction of green area” (8.6%), “the air pollution and stench” (5.8%), and “the pollution of river water” (4.7%) show high rates of increase compared to 2016 and such statistics indicate that they are urgent problems. As for the security issue, one in two citizens in Daejeon think that “the proliferation of security cameras” (48.9%) is indispensable, and after the CCTV expansion “the reinforcement of community police patrol” (18.7%) and “the maintenance of streetlights” (12.2%) follow.

### The 2nd Step of the Needs Map Application: Field Investigation at the Experience Zone Through Layer 4

The Doan district is an area in Doan New Town and Spa Tourist Site (Wonsinheung-dong, Oncheon 1-dong and Oncheon 2-dong) in Yuseong-gu, Daejeon. Yuseong Spa Station of Daejeon Metro Line 1 is located at the Doan district and two avenues (Doan-daero and Gyeryong-daero) cross on this district so that accessibility through public transportations is quite convenient. Also, the Doan district is close to accommodations, shopping towns, and large-scale apartment complexes. Since the user experience elements can be maximized, the Doan district with many infrastructures was finally selected as the experience zone after an advisory board discussion.

**Figure 3:**
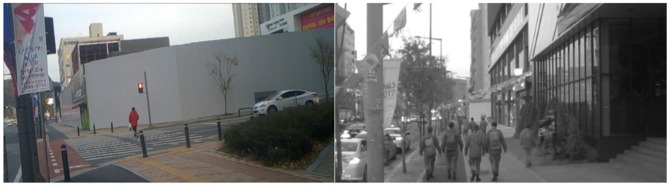
User behavior analysis using image analysis devices.

In this study, image analysis devices (WS-6210 Infrared Thermal Camera) were used for the behavior analysis of the users visiting the Doan district. The field investigation was conducted on November 19, 2017, from 16:30 p.m. to 18:30 p.m. for 2 h. The user behavior survey was conducted only to check the frequency of behaviors and did not infringe on personal portrait rights (see Figure 3).

In the behavior analysis, user's behaviors were separated into 11 pedestrian activity types and the frequency of each behavior type was counted. The behavior types are classified into essential activity (waiting, passage, purchase), selective activity (entertainment/amusement, food/beverage, exercise), and social activity (gathering, conversation, smoking, cultural performance/event). Peculiar features which can be identified visually such as gender, age group, vehicle possession, riding on a motorcycle or bicycle, carrying out a criminal act, or using a mobile phone, or other smart devices were also recorded (Oh and Lee, 2013).

The image data extracted through the image analysis device was coded as “1” for each user's behavior per case using the Microsoft Excel program. In multiple responses, the sum of the cases may be less than the sum of each survey. For example, if four men walked together, they coded four for their gender and one for walking. This is to determine the universal behavior of the user group. A total of 124 cases were extracted through the image analysis. Males visit more frequently, with 92 visits compared to females with 76 visits.

The result of the user behavior analysis shows that the following services are helpful and useful to users who visit the Doan district (An and Kim, 2018):

1. The illegal parking and littering should be solved to provide a safe and comfortable space.
2. It is recommended to provide the service environment with various public information guides, such as living and public transportation.
3. It is necessary to induce various pedestrian activities that enable the affordance of the urban space.

### The 3rd Step of the Needs Map Application: Establishment of Services for the Experience Zone

**Figure 4:**
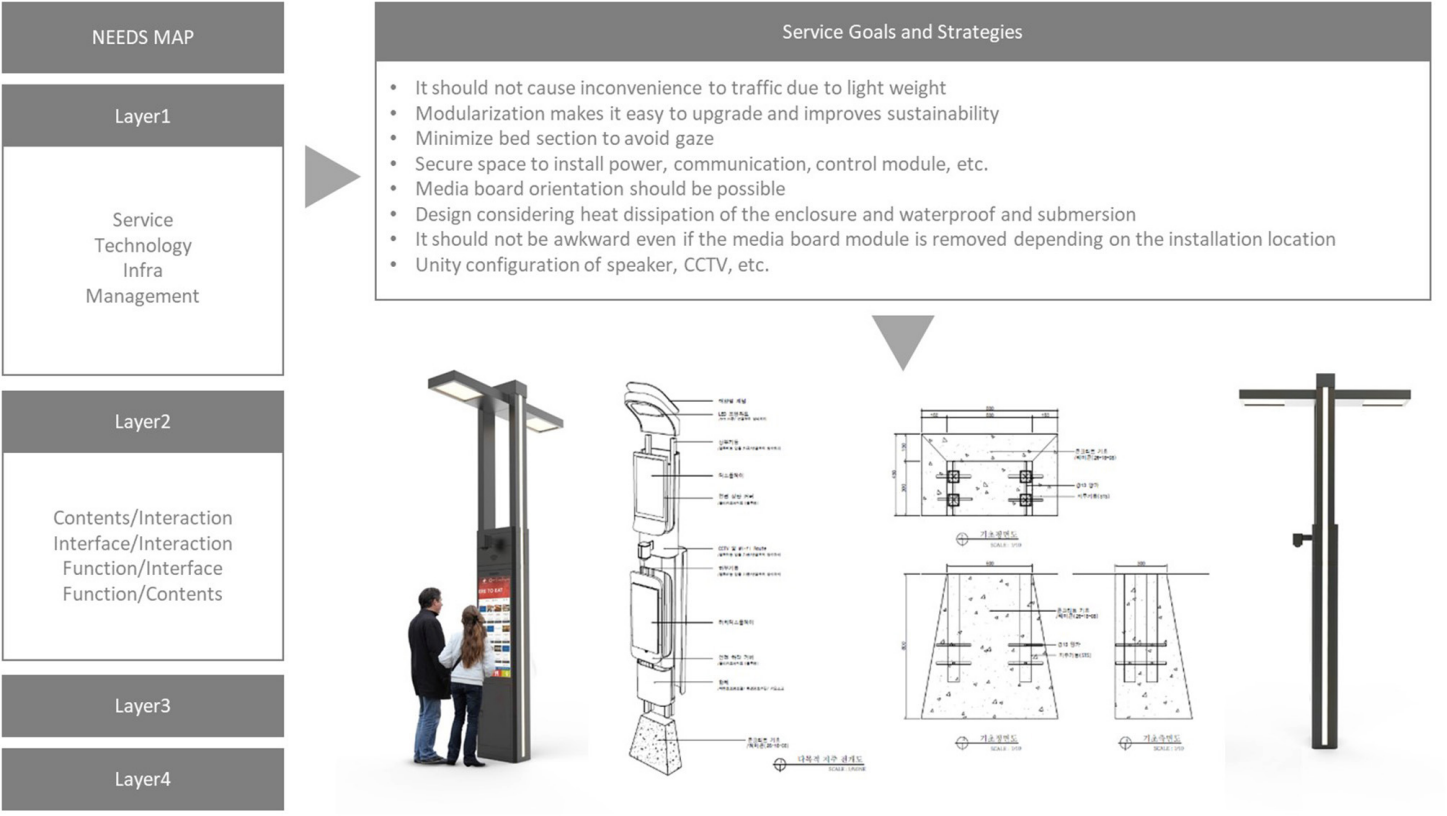
Service goals and strategies of Smart View.

In this step, the entities on the needs map were utilized to develop the smart view service. The developed smart view service can also be installed as an independent street stand based on the module type according to the necessary functions, but our aim is to develop the design plan which is utilizable together with the existing infrastructures. By using the needs map, each smart view function can be described as follows (see Figure 4).

1) Service

Based on items in the service sector of the needs map, the Smart View service was carefully planned. To reflect various needs for user groups we considered the content component, the communication and cooperation within diverse interested parties, and the concept of their role and goal. The content component includes living (supporting the living convenience and safe lives of the citizens), work (activating the local economy of production and enhancing the work efficiency of the related parties), entertainment (activating citizen participation in their local community by specializing local cultural assets), and mobility (securing the convenience, approach, and comfort of pedestrian and transportation methods), etc.

Smart view is a smart streetlight which is modularized to have an assembled structure. It plays a basic role of not only a streetlight but also a security camera. Additionally, it has the environmental monitoring function to measure temperature, humidity, micro dust, and ultraviolet light, and provides public and local information through a kiosk (see Tables 3, 4).

**Table 3:** Functions of smart view according to service.

**Needs Map**			**Service design development**
Category	Specificity	Entity	Smart View Function
Service	Contents/Interaction	Contents	Street light, CCTV Guide: kiosk, Bluetooth, speaker Environmental monitoring: temperature, humidity, fine dust, ultraviolet Guide contents
		Living	Multi-purpose CCTV, street lighting, smart traffic sign, flow management, soil humidity management, smart garbage bin
		Working	Wi-Fi, kiosk, media board
		Playing	Event lighting, Media board
		Moving	Pedestrian safety
		Stakeholders	Bidirectional near field communication
		Agreement	Bidirectional near field communication, remote information measurement data transmission and reception
		Community	Flow management, Soil humidity Management, Smart garbage Can, Kiosk, Multi-purpose CCTV
		Rule	Smart city test bed
		Objective	User experience, efficiency, sustainability

**Table 4:** Main functions of Smart View according to actual service types.

**Type**	**Actual Service**	**Main functions**
Main	PC	Media board and kiosk visual control, event lighting control, street light control
	Communication equipment	Internet modems and routers
	Temperature control Device	Temperature control inside the enclosure
	Power supply Device	Enclosure and module power supplies
	UPS	Power supply at power off
	Earth leakage breaker	Prevent device failure through overvoltage or short circuit
Working living	Bluetooth	Remote information measurement data transmission/reception
	Wi-Fi Route	Create public Wi-Fi___33 zone
	Two way information transfer	Information on museum and exhibition events and facilities is delivered through the user UI
Moving	Crime prevention	Security function of plaza and pedestrian passage
	Emergency call	In case of emergency, call the integrated control center through the emergency call button
Playing	Event Lighting control	Event lighting color control through integrated system
Living	Temperature, humidity, UV measurement	Temperature and humidity, UV measurement
	Fine dust Measure	Fine dust (PM1.0, PM2.5, PM10) measurement

2) Technology

Based on items in the technology sector of the needs map, the technological components of Smart view were carefully planned. To support the two-way communication and management with the citizens in the urban space, sensing and interface technologies were adopted to detect human movement and state, network, and processing for various information exchanges, and security. In particular, the connectability with other hardware, software, and services already used in the city was taken into consideration (see Table 5).

**Table 5:** Functions of smart view according to Technology.

**Needs Map**			**Service design development**
Category	Specificity	Entity	Smart View Function
Technology	Interface/ Interaction	Security Sensing Processing Network Interface	H/W enclosure, beacon, lighting, touch screen, sensing device S/W: Street light control, CCTV control, sensing collection, environmental information display, analysis/statistics Linkage: 119/112 Emergency dispatch disaster emergency response, civil protection and public safety linkage services

3) Infrastructure

Since Infrastructure includes the physical form to realize services into the real space, Smart View was designed in the consideration of the fixed, movable, or variable functional elements. Through modularization, it can be designed into an independent kiosk according to the purpose of necessity, and it can also be designed in the application form to be connected to existing public facilities, such as a streetlight or a bus stop (see Table 6).

**Table 6:** Functions of smart view according to infrastructure.

**Needs Map**			**Service design development**
Category	Specificity	Entity	Smart View Function
Infra	Function/Interface	Built Movable Integration	Pole Module Link

4) Operation Management

Management means not just the service operation, but also the concreteness and possibility of the business model for sustainable services. Thus, the governance establishment for private cooperation and citizen participation, and the management, operation and maintenance of various contents and information were considered. The integrated control to operate all information is important in the view of management, and so the role of the integrated control center is quite essential to information operation for providing the Smart View service and actual management of Smart View (see Tables 7, 8).

**Table 7:** Functions of smart view according to management.

**Needs Map**			**Service design development**
Category	Specificity	Entity	Smart View Function
Management	Function/Contents	Governance public participation Data management Facility management Administration	Integrated control

**Table 8:** Key features of smart view.

**Type**	**Key features**	**Main functions**
Street light function	Illuminance/Light Efficiency/Light Distribution	Meets illuminance, light distribution conditions, and average level of light efficiency
Energy saving	LED	Energy saving and eco-friendly by using LED lighting
	Control	Control required according to the illuminance and color temperature sensor functions
Wireless	Wireless AP	Access point provided, location based service possible (Contents/Program absence is not function)
	Hot Spot	Provides hot spots for wireless Internet connection in DMC
Safety/security	CCTV	Used for security/control of DMS distance
	Speaker	Audio source, real-time voice broadcasting function, two-way communication is not possible
Interactive	Receive information sent	Digital banner, mobile SMS
	Sensing	Possible to direct lighting event through motion sensor
Operation/Maintenance	Integrated Operation center	Ip Intelight remote control, integrated management and LED event lighting
	Upgrade	Newly designed and installed to reduce scalability and add functionality

## Conclusion

In this paper, we recognized the problems of Smart city planning focused on fragmentary technology developments and proposed the needs map as a schematization of theoretical concepts for the Smart city planning in accordance with the framework of the service-technology-infrastructure-management (STIM). This study has important implications for architects, designers, planners, and policy makers who seek to develop creative Smart city services. We confirmed that researchers can thoroughly investigate extensive urban issues or users' needs in a relatively short time through cooperation with interested parties by using the needs map. Researchers can develop or analyse Smart city services through each of the layers and entities on the need map. In other words, service completeness can be improved by double-checking concepts missed in the service realization process.

Furthermore, the users' needs within various social fields with urban issues can be closely understood through the needs map and reflected to the service development. Applying the needs map to not local but whole smart cites requires the management of an immense amount of data which is constantly updated. Needs from variously interested people who are men, women, residents, visitors, officials, etc. are also complicated and extensive. This demonstrates another reason why the needs map is necessary. The needs map proposes a new methodology through which user behaviors are classified, managed, and utilized for needs extraction and further service development creation for Smart cities. The classification of such large-scale data requires several filtering processes of data sets. In algorithms for Big Data Analysis, a filter or kernel is given in the form of a matrix and, in fact, the filtering process is a convolution of the filter with a data set. So, to design filters appropriate to the purpose is very important in data classification. The needs map should permeate these filter matrixes. This filter design for the need map will be our future study. Through our future study, we can analyse the current status of smart services in urban spaces and suggest directions for improving the Smart city services.

**Figure 5:**
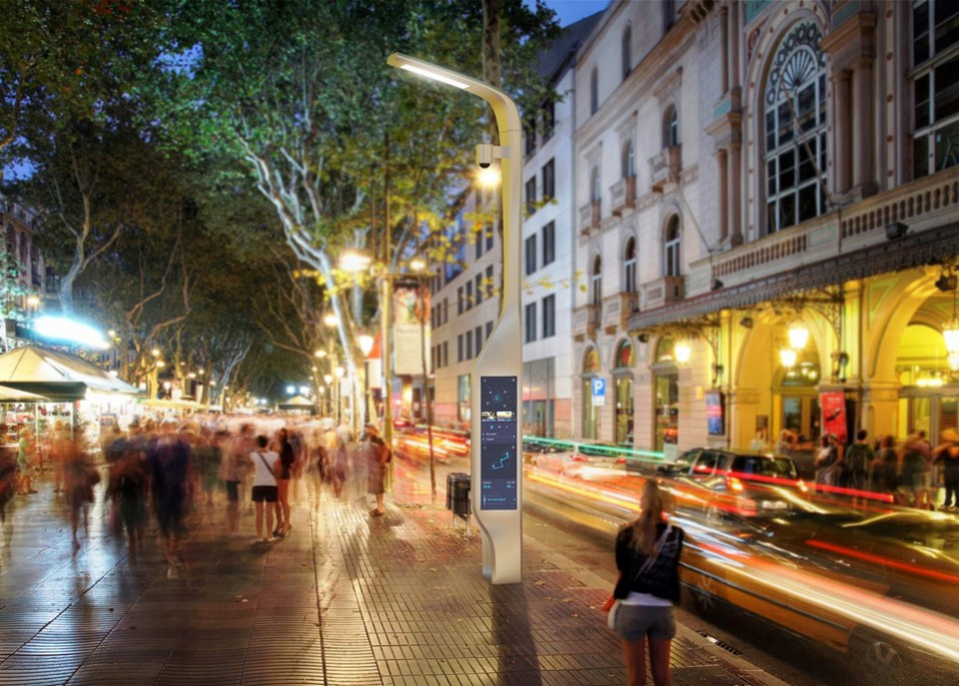
Modeling image of smart view.

To demonstrate the feasibility of the proposed needs map, we provided an application of the needs map to the service design for the Smart city experience zone located in Daejeon, South Korea. We propose the necessity of Smart View on this experience zone (see Figure 5). Smart View is equipped with service functions to address problems raised by the analysis result. However, this application does not verify the sustainability of Smart View in the manuscript because it was not manufactured as a real product. The manufacture of Smart View requires time and money. In fact, it is our long-term future works to demonstrate the usefulness of the needs map and the sustainability of services developed through the needs map.

## Data Availability Statement

All datasets generated for this study are included in the article/supplementary material.

## Author Contributions

SA conceived of the presented idea and developed the theory. SA and SoK performed the computations and verified the analytical methods. SuK helped carry out the Mathematical concepts for analyzing associations between 4 layers of Needs Map. All authors provided critical feedback and helped shape the research, analysis, and manuscript.

### Conflict of Interest

The authors declare that the research was conducted in the absence of any commercial or financial relationships that could be construed as a potential conflict of interest.

## Footnotes

^1^We held five conferences (workshops and expert advisory meetings) for three months from April to September 2017 to derive the need map concept.
